# False data, positive results in neurobiology: moving beyond the epigenetics of blood and saliva samples in mental disorders

**DOI:** 10.1186/s12952-016-0064-x

**Published:** 2016-12-12

**Authors:** A. Cariaga-Martinez, R. Alelú-Paz

**Affiliations:** 1 Laboratory for Neuroscience of Mental Disorders Elena Pessino, Department of Medicine and Medical Specialties, School of Medicine, Alcalá University, Madrid, Spain; 2 Department of Psychiatry, Ramón y Cajal Hospital, IRYCIS, Madrid, Spain

**Keywords:** Epigenetics, Positive results, Peripheral samples, Human brain, Neurobiology, Schizophrenia, Bipolar disorder

## Abstract

Many psychiatric diseases are influenced by a set of several genetic and environmental factors that genetics alone cannot explain. Specifically, in schizophrenia and bipolar disorder the absence of consistently replicated genetic effects together with evidence for lasting changes in gene expression after environmental exposures suggest a role of epigenetic mechanisms in its pathophysiological mechanisms. In this field, the presence of positive results could potentially uncover molecular mechanisms of deregulated gene expression in these complex disorders. In this commentary we have reviewed the positive data obtained over the last 5 years from the scientific literature published in PubMed and we have shown that these results are based on peripheral samples (blood, saliva and other fluids) that do not allow us to obtain reliable and/or valid results, under any circumstances. Finally, we highlight the need to employ human brain samples in the epigenetic study of mental disorders.

## Background

In recent decades we have seen an exponentially increased interest in the role of genetic background in the development of mental disorders. We have observed how scientists have attempted to find an adequate predictor or diagnosis marker by using the genetic approach. However, these efforts have not enabled us to identify a reliable signature from patients’ genes.

Despite these facts, in the last 5 years we are again experiencing the repetition of the cycle, but now it is being applied it to the epigenetic approach. Although a non-negligible percentage of negative results is expectable to be found when using the inductive or deductive rationales, in our daily research work we are observing positive results in virtually all the published data when the epigenetic approach is applied to the psychiatry field.

In this research, we have tried to contrast the hypotheses that lack clear objectives and/or are linked to misconceptions about epigenetics and its applications which can lead to the obtaining of data that, at best is of cases that are not adjusted to the biological reality, and at worst are directly false. To test this, we performed an advanced search in the PubMed database by using straightforward “Medical Subject Heading” terms (MeSH, the controlled vocabulary used to indexing the publications of PubMed database), and subsequently assessed the quality of retrieved data in accordance to the Journal of Citation Report.

The main problem we observed was the careless use of different types of samples when epigenetics was studied. This is a major concern given that the epigenetic is specific from every tissue and, even more, from every single cell type. Also, this issue was noted in published reports from the first quartile, leading us to reflect whether this “quality data” are contributing to more knowledge, or adding more noise in the search of a characteristic epigenetic signature for mental disorders. As a conclusion, we strongly recommend that techniques and approaches that are transversally applied between fields, should be correctly used by taking into account the biological scenario, and to adjust the hypotheses in order to get high quality data that are not only assessed by a position in an index, but also to their adherence to biological facts and reality.

## Main text

As is the case with other scientific disciplines, neurobiology advances through two main processes: induction and deduction. The former moves from the particular to the general, while the latter moves from general statements to particular statements, that is, it begins with a hypothesis and can reach a conclusion only to the extent that the hypothesis can be rejected [1]. Although one can expect a greater number of negative results in experimental designs from the deductive method, due to falsification on testing allowing a hypothesis to be rejected, the analysis of the scientific literature on the neurobiology of psychosis shows a very different picture: too many biomarkers have been presented as major breakthroughs only to be then rapidly dismissed or forgotten. As Mario Maj suggests, this huge mass of evidence is now perceived as a sign of uncertainty and confusion [2].

Epigenetics is no exception; although in recent years, it has acquired a relevant role in the analysis of how genes and environment interplay to develop a mental disorder. The data obtained show a chaotic image inviting us to reflect, and analyze whether this novel scientific approach is not a new victim of the known “publish or perish” philosophy and, specifically, to publish only positive results [3–5]. However, before getting into this analysis, let us define what epigenetics is: Epigenetics studies the heritable information that does not depend on the DNA sequence [6]. In other words, it refers to the interplay between genes and environment that allows the existence of patterns of genetic expression and function, without changing the sequence itself.

Although several events are grouped under the “epigenetic modification” term, the methylation of cytosine residues at the carbon 5 position (5mC) within the dinucleotide CpG is the most widely studied epigenetic modification [7] and, probably, the most interesting for psychiatry given that it represents a dynamic but stable way to regulate gene expression, both in normal or pathological conditions and yet, what do we know about the epigenetic regulation (through DNA methylation) in schizophrenia and bipolar disorder? Despite the large number of positive results in this field, the answer is basically, actually nothing worth knowing.

We can attribute this to several factors: firstly, the lack of reliability and validity of the diagnostic criteria which entails a lack of reliability and validity of the data obtained in the epigenome wide analysis; so in this case, quantity is not as important as quality. Secondly, statistical analyses usually lack rigor, transforming negative results into positive ones. However, in epigenetics, we found a third cause: the kind of sample employed in the experimental designs. Beyond simply trying to understand the causes of psychosis, some researchers focused their efforts in finding new routes by analyzing DNA methylation in blood, saliva or other fluids in order to get putative biomarkers.

With this idea in mind, several observations and empirical data were collected and some results seemed to be promising, as the analyses ranged from studies of methylation pattern in gene promoters, to epigenomic scale tests. However, no consistent results were noted. Furthermore, no replication is still a major handicap in applying epigenetic approach to psychiatry. So, are we on the wrong or on the right track?

Again, we must return to the issue of samples; we know that mental Illness is nothing but brain illness and, therefore, we could think that to study the neurobiology of a specific mental disorder (such as schizophrenia or bipolar disorder) we have to study schizophrenic or bipolar human brain samples. In this way, some scientists claim that brain tissue is extremely hard to obtain and, in fact, epigenetic information acquired from it will not be able to generate any kind of non-invasive (or minimally invasive) diagnosis. The “problem” is that we know DNA methylation is organ-specific; meaning that, muscle tissue has an epigenetic sign that is different from hepatic or brain tissue [8, 9].

Furthermore, epigenetic patterns depend on the cell type and these might represent extreme differences [9]. In fact, these differences are responsible for the role of epigenetics in early differentiation in embryonic cells [10]. Also, epigenetic mechanisms are highly dynamic across cellular populations, for instance, epigenetic processes are essential for maintaining the stemness of progenitor cells as well as the differentiated status of adult tissue [11, 12].

All the aforementioned is in fact, even more complicated when we work with brain tissue: glial cells and several kinds of interneurons and excitatory neurons show characteristic epigenetic marks which differ among them [13]. What's more, this epigenetic signature also varies among neurons from different brain zones [6]. In brief, trying to find epigenetic data in a mixture of cells (even if those come from the same person or the same brain region) will lead to a higher degree of “noise” in the retrieved information. Although there are several mathematical models that try to overcome this [14], as scientists we also need to keep in mind what the biological relevance of the data is, when obtained in this way.

The question arising from this analysis is how neuroscientists face this issue. To answer this question, we perform a search across PubMed in order to get a wide overview focusing on the more recent research in schizophrenia and bipolar disorder.

Although generic terms (as “epigenetic” and “psychiatry”) retrieved thousands of reports, indicating frantic work in this field we use as MeSH Major Topic “schizophrenia” or “bipolar disorder”, and as MeSH Term “DNA methylation”, in order to retrieve the most narrowed data produced over the last 5 years (2011–2016) in scientific papers published in English and indexed within this database. We also restrained our search to papers with data from human samples. We identified 26 papers with the terms “bipolar disorder” plus “DNA methylation” and 56 articles when we used “schizophrenia” plus “DNA methylation” terms.

**Fig. 1:**
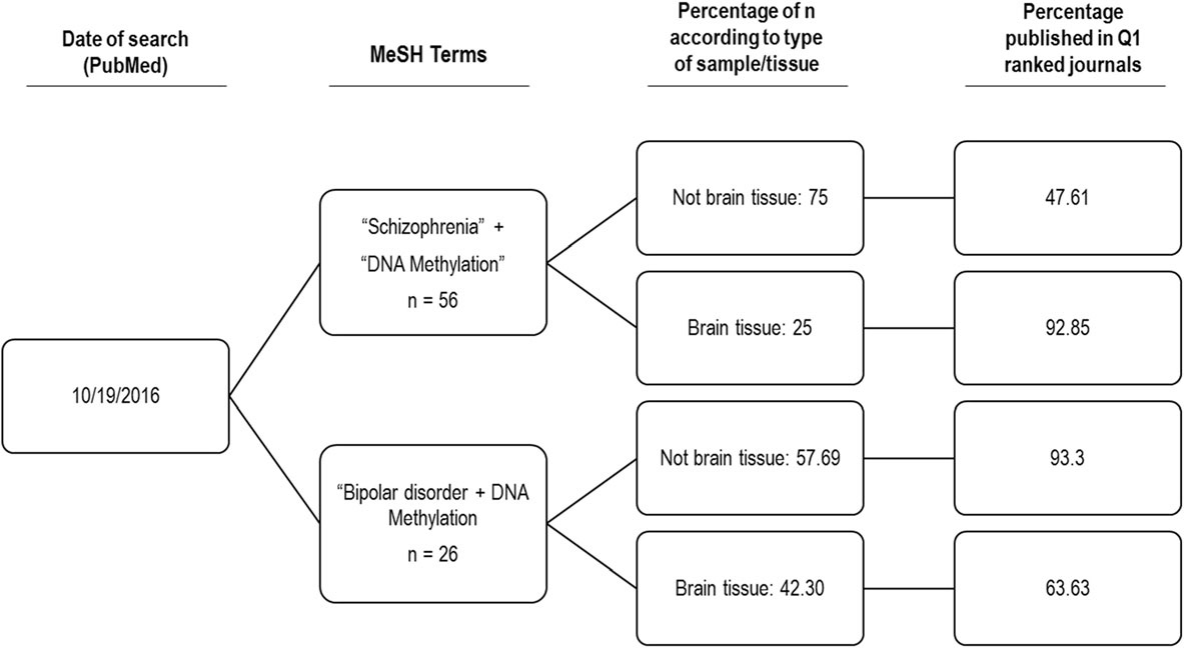
Tree Diagram of advanced search in PubMed database. “Schizophrenia” and “Bipolar disorder” were used as Medical Subject Heading (MeSH) main terms while “DNA methylation” was used as simple term. Branches represents the percentage of published reports when these two keywords were combined. The last branch represent the percentage of published reports in first quartile according to impact factors of the corresponding year of publication (Journal Citation Report—Thompson Reuters). Only data from the last 5 years were included

We find that the samples in around 75 % of the papers under “schizophrenia” plus “DNA methylation” terms, were blood, saliva or other fluids, while the rest of the data were obtained in brain samples. A lower percentage were obtained under the “bipolar disease” plus “DNA methylation” terms: around 58 % of studies claimed to be carried out on blood samples or fluids while the rest were carried out on human brain (see Fig. 1). In schizophrenia, around 93 % of published papers that included brain samples where located in the first quartile of JCR index in psychiatry (vs 63 % in bipolar disorder), percentage similar to articles in the first quartile that employ blood or saliva samples in bipolar case; it is well known that JCR is a recognized index of quality, meaning that these data were peer-reviewed and, potentially, provided with high confidence and quality.

A pessimistic view of this situation would indicate that we are on the wrong track, however, this is not true at all; in schizophrenia 25 % of the papers include brain samples and, in bipolar disorder this percentage rises to 42 %.

And yet we can do more. Firstly, it is easier to use straightforward language when epigenetic findings are detailed. It is strongly recommended to understand that “biomarker” is perhaps not the most adequate word when we study a highly dynamic process such as epigenetics [15]. Basic biology is an exciting field but its results should be translated to clinic with the appropriate caution [16].

Secondly, highlighting the importance of negative results [17]. As long as we stay in the “publish or perish” philosophy, the noise we are dumping on some fields, such as psychiatry, means years or decades of regression in true knowledge. Although it is tempting to find “biomarkers” or “biological signs” for clear diagnosis, we need to keep in mind all the limits of our techniques and approaches, with even more stringency when these approaches are borrowed from other scientific fields that, may not completely fit with ours [18]. In fact, the main risk of data obtained by directly applying the knowledge of some other fields (such as from epigenetic to psychiatry) is the lack of reproducibility [19]. Although this handicap is widely extended in several scientific fields [20], it should represent a major concern in psychiatry as a medical specialty in the frontiers of medicine, biochemistry and pharmacology.

Thirdly, reflection on whether the data obtained from the sample that we decide to use is relevant from a biological point of view. Fourthly, analyze if the number/amount of patient/samples/data strengthen (or weaken) the aforementioned relevance. In answering this, we need to keep in mind that “statistically significant” does not always mean “biologically relevant” [21].

And finally, the simplest measure: to understand that a mental disorder is a brain disorder, such as Griessinger suggested more than 150 years ago and, therefore, we cannot employ blood or saliva samples to study the epigenetics of a mental disorder.

## Conclusion

Epigenetic approach, although tempting as a “holy grail” for explaining what genetics was not able to do in mental disorders, might not be directly applied to psychiatry as if we were working with homogenous cell lines in immunology or with pure mice strains in cancer. Instead, we need to take a step back and to critically reason if our samples, statistical models, diagnose, clinical history of patients, etc., to really help us to contrast our hypotheses. Otherwise, we might publish nice positive results in the first quartile, but we will be contributing to a lesser understanding on what we are trying to shed light.

## Abbreviations

MeSH: “Medical Subject Heading” are terms from the controlled vocabulary used to indexing the publications of PubMed database
